# Occupational noise in the university setting: dosimetric assessment and strategies for exposure reduction

**DOI:** 10.3389/fpubh.2025.1581677

**Published:** 2025-06-04

**Authors:** Leyner Torres-Cobo, Javier Alcázar-Espinoza, Mariuxi Vinueza-Morales, Andrea Muñoz-Tarira, Cristian Vidal-Silva

**Affiliations:** ^1^Facultad Ciencias e Ingeniería, Universidad Estatal de Milagro, Milagro, Guayas, Ecuador; ^2^Facultad de Ingeniería y Negocios, Universidad de Las Américas, Santiago, Chile

**Keywords:** occupational noise, acoustic dosimetry, university auditory health, noise exposure, noise mitigation

## Abstract

**Purpose:**

This study investigates occupational noise exposure in a university setting through dosimetric assessments conducted at Universidad Estatal de Milagro (UNEMI), Ecuador, by analyzing measurements collected in distinct campus areas during two periods, 2017 and 2025.

**Methods:**

Sound pressure levels were measured across selected high-traffic and functional campus areas using standardized equipment in accordance with UNE-EN-ISO 9612:2009.

**Results:**

Measurements revealed an increasing trend in occupational noise exposure across university areas, with 2025 levels substantially exceeding recommended thresholds.

**Conclusions:**

The findings emphasize the critical need for implementing targeted noise mitigation strategies to protect auditory health and preserve academic excellence within higher education institutions.

**Implications:**

Adopting these strategies can significantly reduce occupational health risks and foster a healthier, more effective academic environment. The study proposes comprehensive mitigation strategies tailored to university environments to address these challenges.

## 1 Introduction

Noise-induced hearing loss (NIHL) remains a critical occupational health issue. Continuous exposure to high noise levels has been shown to lead to irreversible auditory damage (1). Workers in industrial sectors such as construction are frequently exposed to noise levels that exceed safe limits (2). These findings underscore the urgent need for effective noise monitoring and control strategies in various work environments.

Occupational noise is a well-documented health hazard that also affects university environments, impacting maintenance staff, faculty, and students, and leading to adverse health outcomes and diminished academic performance. Prolonged exposure to elevated noise levels is associated with irreversible hearing loss, increased stress, fatigue, reduced concentration, and adverse psychological and cardiovascular effects (3, 4).

University environments present unique challenges regarding noise exposure. Sources of noise in these settings include maintenance machinery, HVAC systems, vehicular traffic both on and around campus, and high-density human activities in classrooms, libraries, and recreational areas. Previous research indicates that noise levels in universities can be comparable to, or even exceed, those found in industrial settings (5, 6). Such conditions not only compromise the well-being of the academic community but also threaten the quality of the learning and teaching environment.

Effective classroom communication depends on optimal acoustic conditions (7). Moreover, recent findings indicate that a significant proportion of university students exhibit symptoms of hearing impairment due to high levels of recreational noise exposure (8). This highlights the multifactorial nature of auditory health risks within university environments, combining occupational and recreational exposures.

To provide context, Table 1 summarizes the typical noise levels observed in various university environments, illustrating the range of sound pressure levels that different campus areas might experience.

**Table 1:** Typical sound pressure levels in university campus environments.

**Environment**	**Noise level (dB(A))**	**Comments**
Quiet office	40–50	Minimal background noise
Classroom	50–65	Conversational level
Library	45–55	Low ambient noise
Cafeteria	60–70	Moderate crowd noise
Outdoor campus	70–85	Traffic and human activity
Maintenance area	80–100	Machinery and HVAC systems

The primary objectives of this study are:

1. **Assessment of noise levels:** quantify occupational noise levels.
2. **Health and safety compliance:** assess compliance with international standards.
3. **Mitigation strategies:** propose and evaluate mitigation strategies.

By addressing these objectives, this paper contributes valuable empirical evidence to the literature on occupational safety in higher education, informing institutional decision-makers and health professionals about the need for proactive noise management. Although this study is based on local measurements at UNEMI (9), the trends identified and the proposed mitigation strategies have global relevance for higher education institutions. As universities worldwide face increasing population density and infrastructural expansion, robust noise management policies become crucial to safeguard occupational health and academic performance (4, 10).

The remainder of this paper is organized as follows. Section 2 presents the theoretical framework, reviewing relevant literature on noise exposure and its effects in academic environments. Section 3 details the materials and methods used at UNEMI, including the study design, measurement protocols, and instrumentation. Section 4 reports the results of the dosimetric analysis and safe exposure time calculations. In Section 5, the findings are discussed in the context of existing research and potential factors contributing to increased noise levels are analyzed. Finally, Section 6 concludes the paper with recommendations for noise mitigation strategies and suggestions for future research.

## 2 Background

Occupational noise exposure has been extensively studied across various settings, and its adverse effects on health are well documented in the literature. In academic environments, noise exposure not only poses risks to auditory health—such as progressive hearing loss, tinnitus, and impaired sound discrimination—but also has broader implications for psychological wellbeing and cognitive performance, including increased stress, diminished concentration, and fatigue (1, 11). Moreover, chronic noise exposure can contribute to cardiovascular strain and other physiological effects (12).

In the context of university settings, the sources of noise are diverse. They include mechanical sounds from maintenance equipment and HVAC systems, vehicular and pedestrian traffic, and activities in classrooms and common areas. These factors create an environment where noise levels can fluctuate significantly throughout the day and vary by location, with some regions experiencing levels that rival industrial environments (3, 4). Such conditions pose a unique challenge because they affect a heterogeneous population—from maintenance staff and administrative personnel to faculty and students—each of whom may experience different health and performance-related consequences.

Research has demonstrated that the adverse effects of noise are multifactorial. For example, Golmohammadi and Darvishi (11) provide a systematic review highlighting that noise exposure, when combined with other risk factors, significantly contributes to the global burden of NIHL (13). Such studies form the foundation for understanding the complexity of noise-related health risks in occupational settings. To synthesize the current state of research, Table 2 summarizes the key categories of effects attributed to noise exposure in academic environments. This table highlights auditory and non-auditory outcomes across different target groups, emphasizing the multifactorial nature of noise-induced impairments.

**Table 2:** Categorization of noise-induced effects across university populations.

**Effect category**	**Target group**	**Impact description**
Auditory effects	Maintenance staff, faculty	Progressive hearing loss, tinnitus, and difficulty in perceiving sounds, which may impair communication
Non-auditory effects	Students, faculty	Increased stress, reduced concentration, cognitive fatigue, and diminished academic performance
Physiological effects	All groups	Elevated blood pressure, cardiovascular strain, and higher risks of chronic health conditions

**Figure 1:**
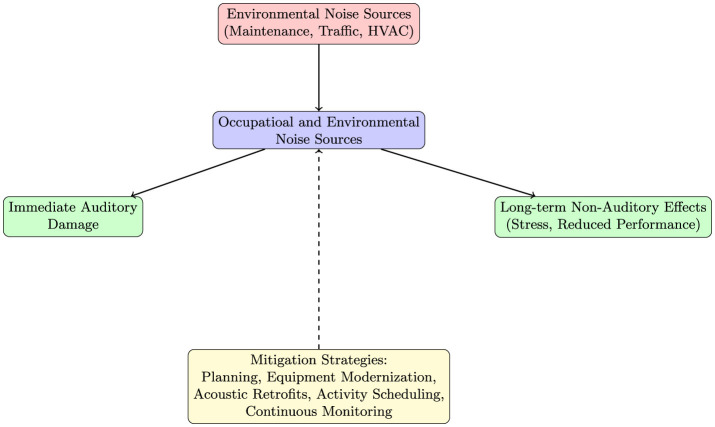
Conceptual model illustrating the pathways through which environmental noise impacts university communities and the role of mitigation strategies in reducing exposure.

Figure 1 presents a conceptual model illustrating the pathways through which environmental noise impacts university communities. The model shows how factors such as inadequate infrastructural acoustics and high population density can exacerbate noise exposure, leading to immediate auditory damage and, over time, to non-auditory health effects. It also highlights the crucial role of mitigation strategies in interrupting these adverse pathways.

Overall, the theoretical framework integrates findings from the literature to establish the complex interrelationships between environmental noise, health outcomes, and academic performance. This framework highlights the urgency of addressing noise exposure in university settings. It sets the stage for subsequent investigation of exposure levels and the evaluation of mitigation strategies in later sections.

## 3 Research design and methodology

### 3.1 Study context, objectives, measurement standards and protocols

This study was conducted at the Universidad Estatal de Milagro (UNEMI) in Ecuador, focusing on the evaluation of occupational noise exposure across key university areas during two distinct periods: 2017 and 2025. The primary objective was to quantify noise levels, assess compliance with international standards, and propose effective mitigation strategies.

Noise dosimetry was conducted following the guidelines established in the UNE-EN-ISO 9612:2009 and NTE INEN-ISO 1996 standards (14). These frameworks provide internationally recognized procedures for determining occupational noise exposure and estimating corresponding risks.

### 3.2 Instrumentation, calibration, and environmental conditions

High-precision noise dosimeters (CESVA DC112 and SPER SCIENTIFIC 850016) were employed for data acquisition. Each device was calibrated before and after every measurement session using standard reference calibrators, ensuring the accuracy and reliability of the collected data. The use of dual instrumentation allowed for cross-validation of the measurements.

Measurements were performed during peak campus activity and periods of lower occupancy to capture the variability in noise levels. The collected data were then processed to obtain Laeq values and compute the maximum permissible exposure times based on the measured noise levels. To illustrate the study design and the sampling sites, Table 3 summarizes the main characteristics of the locations where the measurements were performed.

**Table 3:** Measurement sites and environmental conditions.

**Year**	**Location**	**Coordinates**	**Temp. (°C)**	**Humidity (%)**	**Notes**
2017	Parking area - UNEMI	2°09'05.8”S, 79°36'09.0”W	29.0	60	Low vehicular noise
2017	Sports fields surroundings - UNEMI	2°08'55.6”S, 79°36'08.8”W	28.5	59.3	Moderate activity
2025	Block N - UNEMI	2°07'12.0”S, 79°36'0.0”W	25.9	–	High traffic and dense occupancy

To contextualize the acoustic measurements, environmental parameters such as ambient temperature, relative humidity, and cloud cover were systematically recorded during each measurement session. These contextual data help ensure the reproducibility and reliability of the study outcomes.

### 3.3 Site selection and study design

Measurement sites included high-occupancy academic buildings, administrative offices, student service areas, and open spaces commonly used by the university community. Particular emphasis was placed on Block N, which serves as a hub for teaching, administration, and student interactions, thereby representing one of the university's highest noise exposure zones.

It is important to note that the specific measurement sites assessed in 2017 and 2025 differed due to infrastructural changes and campus development over time. Nevertheless, site selection in each period prioritized areas characterized by high human traffic and activity density, ensuring comparability of exposure profiles across periods.

### 3.4 Occupational noise exposure assessment

The assessment of safe exposure time was based on the calculation of permissible exposure limits derived from Laeq values. The following formula, adapted from ISO 9612:2009, was applied:


(1) $$\begin{array}{l} T=T_{0}\times 2^{\frac{L_{\text{crit}}-L_{\text{meas}}}{\Delta L}} \end{array}$$

where *T* is the maximum allowable exposure time (hours), *T*_0_ is the reference exposure time (typically 8 h), *L*_crit_ is the critical noise level threshold (85 dB(A)), *L*_meas_ is the measured equivalent continuous sound level, and Δ*L* is the exchange rate (3 dB according to ISO standards).

All measurements and calculations adhered to international best practices to ensure the scientific rigor and reproducibility of the study's findings.

## 4 Data analysis and results

### 4.1 Noise levels

Measurements conducted in 2017 recorded Laeq levels of 78.5 dB(A) in the parking area and 82.3 dB(A) near the sports fields. By contrast, in 2025, Laeq levels at Block N reached 90.93 dB(A), significantly exceeding the recommended occupational threshold of 85 dB(A) (14). This increase indicates a worsening auditory environment on campus, potentially reducing permissible exposure times and increasing the risk of noise-induced health issues among students and staff.

### 4.2 Safe exposure time

**Figure 2:**
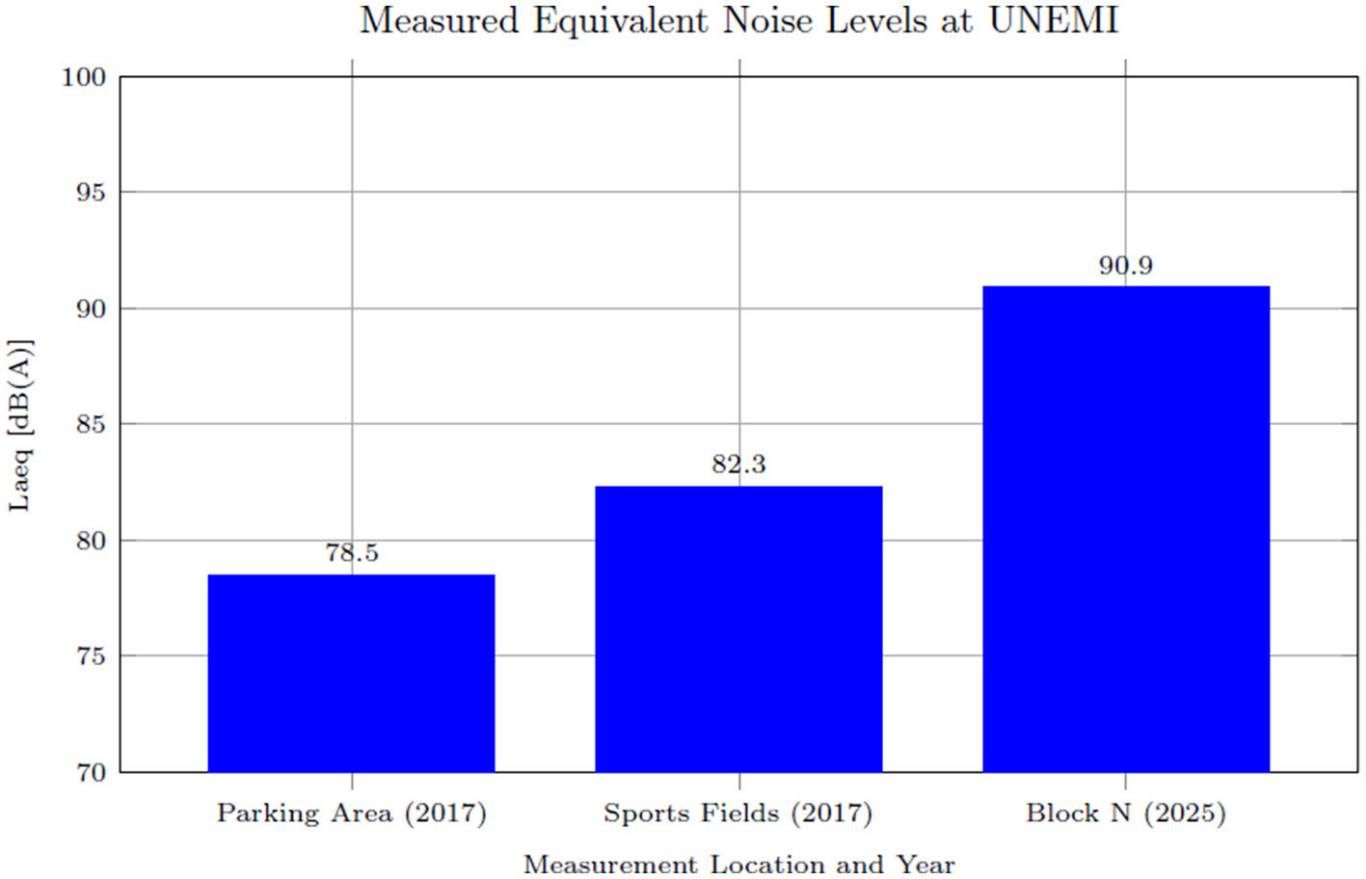
Laeq values measured in different university areas during 2017 and 2025.

Figure 2 illustrates the Laeq values recorded across different university areas during 2017 and 2025, showing a substantial increase in noise exposure over time. Based on these measurements, Table 4 summarizes the key descriptive statistics, including minimum, median, maximum, and Laeq values, along with the estimated safe exposure times for each site.

**Table 4:** Descriptive statistics of measured noise levels.

**Measurement site**	**Min (dB)**	**Median (dB)**	**Max (dB)**	**Laeq (dB)**	**Safe exposure time (h)**
Parking area - UNEMI (2017)	72.4	77.6	81.3	78.5	~12.0
Sports fields - UNEMI (2017)	74.2	81.5	85.6	82.3	~8.0
Block N - UNEMI (2025)	86.7	90.1	94.8	90.93	~3.5

### 4.3 Mitigation strategies

The observed reduction in safe exposure times highlights the urgent need for comprehensive noise mitigation strategies at UNEMI. Accordingly, the following measures are recommended to address escalating occupational noise exposure in university environments:

- **Engineering controls:** redesign work and learning spaces with improved acoustic insulation and upgrade maintenance equipment to quieter models (15).
- **Administrative controls:** implement work rotation schedules and strategically organize academic and administrative activities to minimize individual exposure durations (16).
- **Personal protective equipment:** provide and mandate the use of certified hearing protection devices for staff and students operating in high-noise areas (17).
- **Awareness and training:** conduct regular training sessions and awareness campaigns on the risks associated with noise exposure and the correct usage of protective equipment (18).

Although these recommendations were formulated based on measurements conducted at UNEMI, they are broadly applicable to higher education institutions worldwide. Implementing such strategies can significantly contribute to improving occupational health standards and enhancing the overall academic environment (10).

## 5 Discussion

The impact of high noise levels is not limited solely to hearing loss. Picard et al. (19) found a significant association between noise exposure and increased work-related accidents. In addition, excessive noise can contribute to vocal strain, as highlighted by Rantala et al. (20), who observed a link between ergonomic risk factors and vocal symptoms in educators. Moreover, Shield and Dockrell (10) demonstrated that both environmental and classroom noise adversely affect academic performance in primary school children, suggesting that noise control is essential in educational and occupational settings.

The Laeq values measured in 2025, particularly at Block N, exceeded the recommended threshold of 85 dB(A), resulting in limited safe exposure times of ~3.5 h. These findings have profound implications for the health and wellbeing of the university community. Noise measurements were conducted in different campus areas during 2017 and 2025, each reflecting the environmental exposure typical of that period, without implying a direct longitudinal comparison. Although every effort was made to select representative high-traffic zones for each period, infrastructural changes and campus development between 2017 and 2025 posed inherent challenges to ensuring identical site assessments. Variations in environmental context, such as surrounding vegetation and urban layout, might partially account for the discrepancies observed. While calibration procedures and repeated measurements helped to ensure data reliability, some degree of measurement uncertainty is inherent in any field study. Future studies should prioritize longitudinal monitoring at fixed locations to strengthen temporal comparability and trend analysis.

Thus, the results illustrate variability in occupational noise levels across the university campus and underscore the need for location-specific mitigation strategies. The selected sites represent typical scenarios of campus activity and were chosen to reflect changes in environmental noise conditions over time.

The elevated noise exposure observed in 2025 can be attributed to several factors. First, the expansion of campus infrastructure and increased population density have likely contributed to higher ambient noise. Second, aging maintenance equipment and inadequate noise control measures may have exacerbated the problem. Third, post-pandemic campus dynamics, including increased vehicular and pedestrian traffic, have further amplified noise levels. Similar trends have been reported in previous studies (5, 6), emphasizing the global nature of this challenge in academic settings.

Urban soundscape studies reveal that high population density can amplify ambient noise levels in educational environments (21). In addition, exposure to elevated noise levels has been linked to reduced cognitive performance and impaired short-term memory in children (22).

Table 5 summarizes the potential contributing factors identified in this study and suggests corresponding mitigation strategies. Implementing these measures could help to curb the increasing noise levels and safeguard the auditory health of staff, faculty, and students.

**Table 5:** Contributing factors and proposed mitigation strategies.

**Contributing factor**	**Proposed mitigation strategy**
Increased campus density	Implement effective campus planning and manage pedestrian/vehicular traffic
Deterioration of maintenance equipment	Regularly update and maintain equipment; adopt quieter machinery
Inadequate acoustic design	Retrofit existing buildings with sound-absorbing materials and enhance architectural acoustics
Increased post-pandemic activity	Develop policies to control noise during peak periods; schedule noise-sensitive activities during quieter times
Lack of continuous noise monitoring	Establish a continuous acoustic monitoring program to detect and address noise hotspots

The adverse health effects of noise exposure extend beyond auditory damage, affecting overall wellbeing (3). It is essential to adhere to international guidelines and implement noise reduction measures to mitigate occupational risks (4). Furthermore, automated noise assessment models have proven effective in evaluating exposure levels in various occupational environments (6). Continuous exposure to high noise levels can also induce oxidative stress and increase cardiovascular risk, highlighting the need for prompt intervention (23).

Future evaluations should incorporate a more comprehensive statistical analysis of the noise distribution, including minimum, maximum, and median values, to better capture exposure variability. Although noise levels in areas such as the parking lot and sports fields may have increased over time, direct longitudinal verification was beyond the scope of this study.

In summary, the elevated noise levels observed at UNEMI pose a significant risk to occupational health, academic performance, and overall quality of life. Immediate interventions combining infrastructural improvements, policy initiatives, and continuous noise monitoring are crucial. Future research should further refine these mitigation strategies, evaluate their effectiveness longitudinally, and extend similar assessments to other higher education institutions to determine the global scope of this emerging issue.

## 6 Conclusions

This study has assessed occupational noise levels at Universidad Estatal de Milagro (UNEMI) through measurements conducted in distinct high-activity areas in 2017 and 2025. These measurements provide insight into the variability in noise exposure levels across the campus. The dosimetric analysis revealed that the equivalent sound pressure level (Laeq) in 2025 reached 90.93 dB(A) at Block N–substantially exceeding the internationally recommended threshold of 85 dB(A). Measurements recorded Laeq values of 78.5 dB(A) and 82.3 dB(A) in 2017 at other areas of the campus. The Laeq level measured at Block N in 2025 (90.93 dB(A)) corresponds to a reduced safe exposure time of about 3.5 hours, increasing the risk of noise-induced hearing loss, stress, and adverse impacts on academic performance.

The implications of these findings are multifaceted: (i) **Health risks:** The reduction in safe exposure time highlights a heightened risk for auditory damage among maintenance staff, faculty, and students. Prolonged exposure to such high noise levels will likely contribute to hearing loss, tinnitus, and other health issues. (ii) **Academic impact:** Increased noise levels can compromise the learning environment by affecting concentration and overall academic performance, impacting both teaching quality and student outcomes. (iii) **Urgent need for intervention:** The data indicate that immediate and effective noise mitigation measures are essential to safeguard the wellbeing of the university community.

Based on the results of this study, the following recommendations are proposed:

1. **Continuous noise monitoring:** establish a continuous acoustic monitoring system across the campus to identify and manage noise hotspots promptly.
2. **Infrastructure enhancements:** retrofit existing structures with sound-absorbing materials and modernize outdated maintenance equipment to reduce ambient noise levels.
3. **Policy implementation and awareness campaigns:** develop and enforce noise control policies and conduct regular awareness campaigns to educate the university community about the health risks of excessive noise exposure.
4. **Further research:** future studies should evaluate the effectiveness of implemented mitigation strategies and explore additional interventions that may be applied to similar academic environments globally.

The elevated noise levels observed at UNEMI reflect a broader challenge faced by modern academic institutions. Proactive and comprehensive noise management measures are crucial for protecting auditory health and maintaining an optimal environment for teaching and learning.

## Data Availability

The original contributions presented in the study are included in the article/supplementary material, further inquiries can be directed to the corresponding author.
